# The Association of C-Mill With Traditional Physical Therapy Rehabilitation in a Multiple Sclerosis Patient: A Case Report

**DOI:** 10.37825/2239-9747.1061

**Published:** 2024-12-04

**Authors:** Emanuele Lo Voi, Marina Garofano, Agnese Gugliandolo, Placido Bramanti, Mariaconsiglia Calabrese, Luigi Bibbò, Gianluca Fimiani, Michele Senatore, Francesco Corallo, Emanuela Mazzon, Alessia Bramanti

**Affiliations:** a IRCCS Centro Neurolesi Bonino Pulejo of Messina, Italy; b Department of Medicine, Surgery and Dentistry “Medical School of Salerno” University of Salerno, Italy; c Department of Medicine, Surgery and Dentistry, University of Cagliari, Italy; d Università degli Studi eCampus, Novedrate, Italy; e DICEAM, Department of Cvil, Environment, Energy and Material Engineering, Reggio Calabria, Italy; f Department of Informatics, University of Salerno, Italy; g Ordine dei Fisioterapisti of Salerno, Italy; h Department of Innovative Technologies in Medicine and dentistry, University of Chieti, Italy

**Keywords:** Multiple sclerosis, Robotic rehabilitation, C-Mill gait assessment

## Abstract

A 55-year-old woman with Multiple Scleroris (MS) was referred to undergo a course of conventional physical therapy and cognitive robotic rehabilitation. The neurological examination revealed ataxic gait, dysmetria, nystagmus, cognitive impairment of attention and memory, weakness in the four limbs. To improve balance and walking skills, 20 gait and balance rehabilitation sessions was prescribed, based on conventional physiotherapy and virtual reality. The results were evaluated with balance and walking tests and with objective test C-GAIT. This case report shows improvements in gait and balance in aMS patient obtained with a rehabilitation program that associated C-MILL with traditional physical therapy.

## 1. Introduction

Multiple sclerosis (MS), also called plaque sclerosis, disseminated sclerosis, or polysclerosis, is a chronic autoimmune demyelinating disease that affects the central nervous system causing a wide spectrum of signs and symptoms. It was first described by Jean-Martin Charcot in 1868. The disease can occur with a wide range of neurological symptoms and can progress to physical and cognitive disability. Multiple sclerosis can take several forms, including a relapsing-remitting, primary progressive, and secondary progressive forms. The disease has a prevalence ranging from 50 to 300 cases per 100 000 individuals [1]. The MS hallmark is represented by focal plaques that are the areas of demyelination that are present in the white and gray matter, with the loss of oligodendrocytes, blood–brain-barrier destruction, infiltration of inflammatory cells and neuroinflammation [2,3]. The damage is mediated by the immune system, that cause an immune-mediated myelin destruction. In the tissues and serum of MS patients, antibodies directed against CNS myelin proteins, lipids, and carbohydrates were found [4].

Although the mechanism by which the disease occurs is well studied, the exact etiology is still unknown. The various theories propose both genetic and infectious causes; moreover, correlations with environmental risk factors have been highlighted [1].

Most likely, MS is caused by a combination of genetic, environmental and infectious factors and possibly by other factors such as certain vascular diseases [5]. Epidemiological studies of the disease have provided indications on the possible causes. Theories try to combine known data into plausible explanations, but none of these have been conclusive.

Nowadays there is no known cure. Some pharmacological treatments are available to avoid new attacks and prevent disabilities [6]. The prognosis is difficult to predict and depends on many factors, while life expectancy is about 7 years lower than that of the healthy population [7]. The most common symptoms include gait deficits, balance and coordination impairments, fatigue, spasticity, dysphagia and an overactive bladder, so conventional physical therapy can maintain or improve balance, mobility and reduce impact of spasticity [8].

Technological development offers new possibilities in the field of neurorehabilitation, for treatment, diagnosis and monitoring of progress. In medical rehabilitation, virtual reality has brought new solutions for a variety of pathologies. However, robotic rehabilitation and virtual reality (VR) may be more efficacious in improving MS symptoms, including balance and gait disorders [9]. Neurorehabilitation therapeutic approaches aim to alleviate symptoms and improve the quality of life through promoting positive immunological transformations and neuroplasticity. Here, we reported a case of a 55-year-old woman affected by MS with difficulties in walking and balance that showed improvements with the association of traditional physical therapy and exergame VR on C-MILL VR + (Motek Medical B.V., Houten, Netherlands).

## 2. Case report

The patient is a 55-year-old woman affected by MS, with onset in 1994 with diplopia and paraesthesia, diagnosed at the Civic Hospital of Palermo after MRI of the brain.

The patient works as a teacher, is married with a daughter, is independent in walking, but can no longer drive. She does not smoke or drink alcohol, is 165 cm tall and weighs 55 kg (Body Mass Index 20). She showed a fair mobility of the lower limbs, with greater compromise on the left. She did not show major sensory deficits and showed a discrete trunk control and sitting balance reactions.

In 2012, she started therapy with Copaxone due to gait disturbances. Subsequently, she changed therapy with Fingolimod (Gilenya), interrupted due to the appearance of basalioma in the sternal region. In January 2015 she performed left mastectomy for breast carcinoma. Thereafter she began therapy with Tecfidera, which she still continues today.

In 2017 she was hospitalized at IRCCS Centro Neurolesi Bonino Pulejo, for a rehabilitation cycle. In 2019 she was hospitalized for rehabilitation at the S. Lucia Hospital in Rome.

In 2021, the patient came again to IRCCS Centro Neurolesi Bonino Pulejo, for a new rehabilitation cycle. The neurological examination before rehabilitation revealed cognitive impairment of attention and memory, weakness in the four limbs with greater impairment on the left.

The subject comes to our attention alert and cooperating, with a slight deficit of strength in the upper limbs especially on the left with motor impediment in the hand. In the lower limbs she has a fair mobility with greater compromise on the left. There are no major sensory deficits. She showed a discrete trunk control and sitting balance reactions and she was able to maintain autonomously station erected on an enlarged base. The Romberg test was positive with multiple fluctuations. She showed autonomous walking, ataxic gait with a tendency to right lateral drive.

### 2.1. Assesment of balance and gait

Following the clinical examination, the patient underwent basic assessment of balance and gait at T = 0 (rehabilitation taking over).

For the assessment of balance abilities, an expert physiotherapist administered the Berg Balance Scale (BBS) [10], a commonly used and well validated measure of functional balance. To assess the risk of falling, the Falls Efficacy Scale-International [11] and Tinetti Scale [12] were administered.

The 10 mt walking test scales and the 6 min walking test were administered for the assessment of walking skills.

Furthermore, for a more objective evaluation, an automatized and standardized assessment, called CGAIT, was administered, carried out directly on the C-MILL VR + device, which is used to view the functional performance of walking. It takes 18 min and includes 7 different exercises:

- Visually guided steps;
- Tandem walking;
- Avoidance of obstacles;
- Slalom walk;
- Speed adjustments;
- Reaction to unexpected perturbation.

Each exercise must be performed twice at different difficulty levels: once at an easy level (level 2) and once at a more difficult level (level 4).

**Fig. 1:**
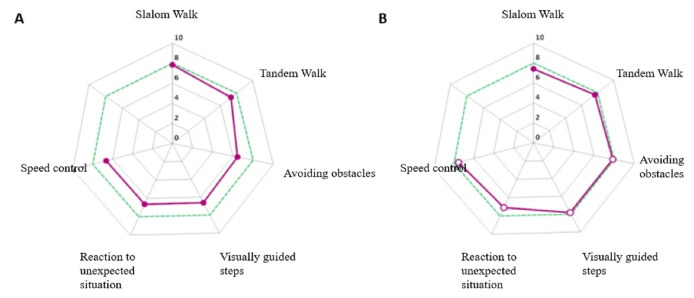
Spider diagram representing the patient’s average score on each exercise performed (purple continuous line) at T = 0 (A) and after four weeks of treatment (B).

At the end of the evaluation, the score of each exercise is presented by means of an innovative spider diagram (Fig. 1). The C-Gait score for each task was evaluated as follow: Level × 2 × Performance (%)/100. The composite score was an average score based on average performance over the six walking adaptability tasks at the higher level of difficulty. The composite score ranged from 0 (poor performance) to 8 (excellent performance).

The assessment was performed at a comfortable speed to facilitate comparison with the walk of daily life outside.

Final assessment was done at T = 1 (four weeks of treatment).

### 2.2. Rehabilitation program

After the basic evaluation, the patient began a rehabilitation program of 20 sessions based on a first part of traditional physical therapy and a second part consisting of exergame VR on C-MILL VR +. This program lasted 4 weeks, with five sessions per week, each session last 120-minute.

The first part of the 60-minutes rehabilitation session included: 20 min of stretching and passive mobilization in the supine position, 20 min of assisted exercises and resistance exercises, 20 min of exercises for trunk control, balance exercises with support of stabilometric and proprioceptive platforms and exercises for the improvement of walking [13,14].

The second part of the 60-minutes rehabilitation session included exercises carried out with the CMILL VR + device. The C-MILL VR + is a treadmill intended to evaluate human gait and balance and to train patients’ gait and balance using treadmill motion, augmented reality and VR. The main features of this machine are:

- Effective functional gait therapy;
- Therapy Based on the Principles of Motor Learning;
- Fun and enjoyable therapy;
- Objective evaluation;
- Efficient therapy;
- Training on the treadmill.

Feasible exercises using the c-mill include the following:

- Bricks (the subject, while walking, must step on bricks, projected into the treadmill, placed at different lengths in order to improve the length and width of the step);
- Obstacle (the subject, while walking, must cross obstacles of different sizes, projected into the treadmill);
- Speed adjustment (the patient must adjust their walking speed based on the speed variation of the treadmill);
- Tandem (the patient must walk in tandem while maintaining the trajectory within an area projected into the treadmill);
- Slalom (the patient must walk by performing slaloms while keeping the trajectory within an area projected into the treadmill);
- Traffic Jam (the patient must remain in monopodalic equilibrium to allow the passage of a car projected on the front screen);
- Italian Alps (the patient, while walking, must move left and right to collect the ingredients to complete a pizza, avoiding obstacles and cannot take the ingredients he has already collected).

All sessions were followed by an expert physiotherapist, who verified the correct execution of the exercises and the patient’s general condition, providing verbal instructions to help the patient maintain correct posture during walking, physical exercise and VR. The patient’s safety is, also, ensured by wearing a standard safety harness, as can be seen in the Picture 1.

### 2.3. Outcomes

The main outcomes analyzed are represented by the rating scales for gait and balance.

In the last evaluation session, the patient underwent the same tests as at baseline.

**Graph 1:**
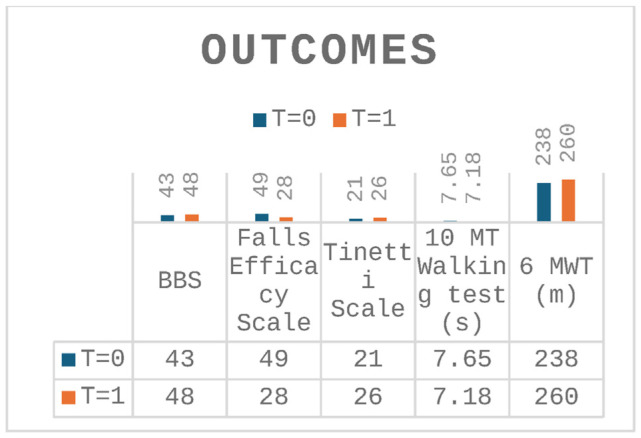
Changes in scores from baseline to 4 weeks.

The assessment at T = 1 showed a marked improvement in all domains analyzed at T = 0 (walking and balance skills) (Table 1 and Graph 1).

Improvements can be observed in all outcome measures analysed, and improvements in gait and balance demonstrated by BBS (greater than 3 points considered to be the minimal important change in the MS patients) [15], Tinetti Scale (greater than 4 points considered to be the minimal important change) [16], 10 mt walking test scales and the 6 MWT, which is 22 m (greater 19.7 m considered to be the minimal important change in the MS patients) [17], were associated with a decrease in the fear of falling as demonstrated by the Falls Efficacy Scale-International, that showed the best improvements, shifting from high to moderate concern and about falling [18].

Also, C-GAIT assessment was administered, carried out directly on the C-MILL VR + device. The scores obtained before, and the end of the rehabilitation program indicated an improvement in walking and balance skills (Table 2; Fig. 1).

C-GAIT assessment is a more objective quantitative tool for evaluating walking adaptability with different tasks in augmented reality, in contrast to the traditional scale evaluation. The patients showed improvements in visually guided steps, tandem walking, avoidance of obstacles, speed adjustments, reaction to unexpected perturbation. No improvement was found in the task slalom. Indeed, changes in direction represent an important aspect of mobility. The maintenance of balance during and after turning involves the complex coordination of sensory and motor systems needed to stabilize and reorient the body towards the new direction of travel [19]. For this reason, slalom represents the hardest task. The score of each exercise is also represented as spider diagram (Fig. 1), that shows visually the improvements recorded.

**Fig. 2:**
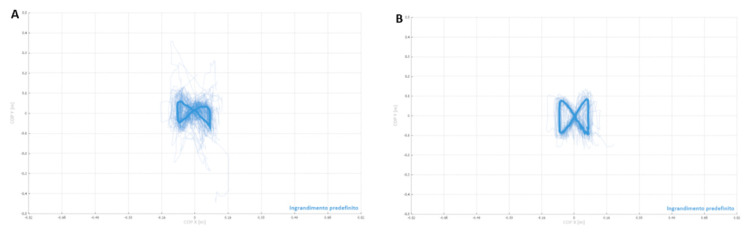
Butterfly shape The butterfly diagram shows the trajectory of the subject’s hundredth pressure at T = 0 (A) and after four weeks (B). The bold line represents the average trajectory of the pressure hundred, while the lighter lines represent the single walking cycles.

During walking on the C-Mill the Centre of Pressure (CoP) is registered during stance phase and the trajectory of the CoP give as a result the shape of a butterfly. The shape of the butterfly provides features of the gait pattern. In particular, the butterfly shape (Fig. 2) of the patient at T0 is asymmetric (Fig. 2-A), while at T1 is more symmetric (Fig. 2 – B) and similar to a healthy one, supporting the improvements in gait and balance reported by C-GAIT.

## 3. Discussion

Motor dysfunctions represent a common symptom in MS, with a severe compromission of gait, coordination, and balance [20]. As a consequence, a reduction of patient quality of life is reported, with compromission of patient’s daily living activities and a worsening of their psychological status. In particular, the main gait abnormalities in MS patients include a reduction in velocity and stride length, an increase in double-limb support time, and gait asymmetries. However, one of the main disabling symptoms is balance impairment, associated with reduced mobility and independence. Impaired balance and gait have also been associated with an increased risk of falling [21]. The Falls Efficacy Scale International questionnaire is the most commonly used scale to measure the fear of falling, that affect the majority of MS patients. This fear decreases self-confidence towards own physical and social abilities. The patient is constantly afraid of falling, then isolate themselves from physical activities and social contacts, which can severely restrict their quality of life. In turn, physical inactivity increases muscular and cognitive decline, leading the patient to the loss of confidence in performing daily activities and of its independence [18]. Then, the improvements in balance and gain increase the self-confidence of the MS patient, and as a consequence improve also his self-confidence and psychological status.

Rehabilitation programs for balance and gait can led to improvements in MS patients. Rehabilitation showed promising results as part of the treatment for MS. In particular, new technologies, such as robotic rehabilitation and VR are emerging to ameliorate some MS symptoms, such as balance and gait disorders [9], as we have seen from the case report presented.

The virtual interactive experience increases the self-confidence of the MS patient. Indeed, the patient can play an active role in similar environments with an increased knowledge and self-consciousness, increasing the motivation, the interest and the development of the motor and cognitive components. Trials demonstrated a positive effect of VR in MS patients for quality of life and balance compared to traditional rehabilitation [22].

Moreover, studies support the main role of VR associated to robotic rehabilitation in improving motorand cognitive performances in MS patients [23].

C-MILL application was shown to exert benefits in other neurological diseases, such as Parkinson’s disease and stroke [24–27]. However, there are no data about C-MILL application in MS patients.

## 4. Conclusion

MS is characterized by motor impairment. Rehabilitation can ameliorate MS motor dysfunctions. This case report highlights gait and balance improvements in a MS patient who followed a rehabilitation program that associated VR on a C-MILL VR + device with traditional physical therapy. The ameliorations were demonstrated both with traditional scale evaluation and C-GAIT scores. The results obtained by the MS patient after the cycle of 20 mixed traditional and innovative technology treatments are better than those she obtained from all previous cycles of rehabilitation performed only in the traditional mode. To our knowledge this is the first case that reported improvements in motor function in a MS patient using C-MILL. Therefore, we suggest that the combination of VR and traditional physical therapy may be a better option for the rehabilitation of MS patients. The promising results obtained in our case report could, in the future, be further confirmed by a pilot study, also aimed at investigating the feasibility and safety of a rehabilitation programme involving the use of innovative technologies for MS patients.

## Figures and Tables

**Picture 1:**
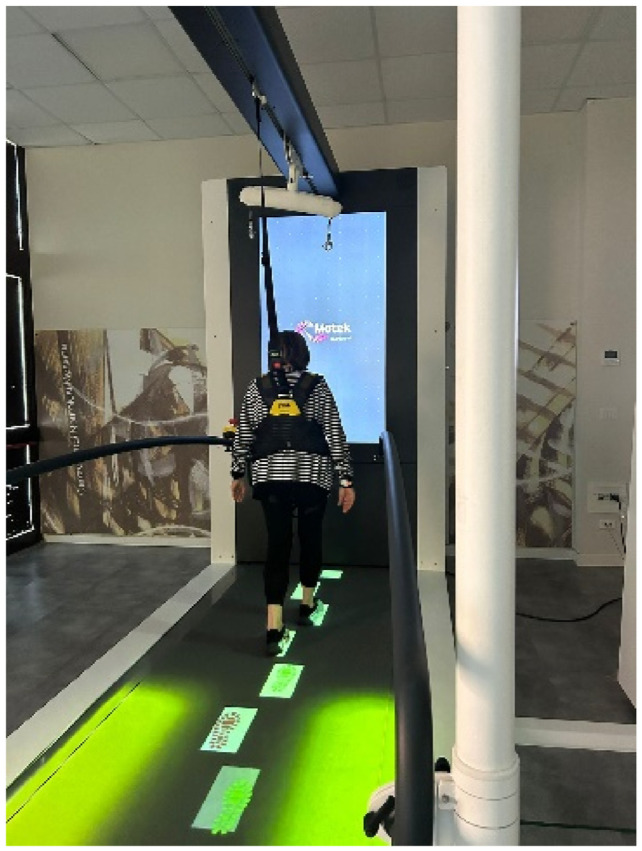
Patient performing walking exercises with the c-mill, with safety harness.

**Table 1:** Outcomes for each test at baseline and at the end of rehabilitation program.

Outcomes	T0	T1
BBS	43/56	48/56
Falls Efficacy Scale	49/64	27/64
Tinetti Scale	21/28	26/28
10 MT Walking test (s)	7.65	7.18
6 MWT (m)	238	260

**Table 2:** C-GAIT score assessment for each task at level 4 at baseline and at the end of rehabilitation program.

C-Gait assessment (score)	T0	T1
Visually guided steps	6.64	7.84
Tandem walking	7.28	7.68
Avoidance of obstacles	6.48	7.84
Slalom walk	7.84	7.44
Speed adjustments	6.64	7.52
Reaction to unexpected perturbation	6.64	7.12
C-Gait composite score	6.92	7.57
